# Comparison of machine learning techniques to predict all-cause mortality using fitness data: the Henry ford exercIse testing (FIT) project

**DOI:** 10.1186/s12911-017-0566-6

**Published:** 2017-12-19

**Authors:** Sherif Sakr, Radwa Elshawi, Amjad M. Ahmed, Waqas T. Qureshi, Clinton A. Brawner, Steven J. Keteyian, Michael J. Blaha, Mouaz H. Al-Mallah

**Affiliations:** 1 King AbdulAziz Cardiac Center, Ministry of National Guard, Health Affairs, King Abdulaziz Medical City for National Guard - Health affairs, King Abdullah International Medical Research Center, King Saud bin Abdulaziz University for Health Sciences, Department Mail Code: 1413, P.O. Box 22490, Riyadh, 11426 Kingdom of Saudi Arabia; 2 Princess Nourah bint Abdulrahman University, Riyadh, Saudi Arabia; 3 Wake Forest School of Medicine, Medical Center Boulevard, Winston-Salem, NC USA; 4 Division of Cardiovascular Medicine, Henry Ford Hospital, Detroit, MI USA; 5 Johns Hopkins University, Baltimore, MD USA

**Keywords:** FIT (Henry ford ExercIse testing) project, All-cause mortality, Machine learning

## Abstract

**Background:**

Prior studies have demonstrated that cardiorespiratory fitness (CRF) is a strong marker of cardiovascular health. Machine learning (ML) can enhance the prediction of outcomes through classification techniques that classify the data into predetermined categories. The aim of this study is to present an evaluation and comparison of how machine learning techniques can be applied on medical records of cardiorespiratory fitness and how the various techniques differ in terms of capabilities of predicting medical outcomes (e.g. mortality).

**Methods:**

We use data of 34,212 patients free of known coronary artery disease or heart failure who underwent clinician-referred exercise treadmill stress testing at Henry Ford Health Systems Between 1991 and 2009 and had a complete 10-year follow-up. Seven machine learning classification techniques were evaluated: Decision Tree (DT), Support Vector Machine (SVM), Artificial Neural Networks (ANN), Naïve Bayesian Classifier (BC), Bayesian Network (BN), K-Nearest Neighbor (KNN) and Random Forest (RF). In order to handle the imbalanced dataset used, the Synthetic Minority Over-Sampling Technique (SMOTE) is used.

**Results:**

Two set of experiments have been conducted with and without the SMOTE sampling technique. On average over different evaluation metrics, SVM Classifier has shown the lowest performance while other models like BN, BC and DT performed better. The RF classifier has shown the best performance (AUC = 0.97) among all models trained using the SMOTE sampling.

**Conclusions:**

The results show that various ML techniques can significantly vary in terms of its performance for the different evaluation metrics. It is also not necessarily that the more complex the ML model, the more prediction accuracy can be achieved. The prediction performance of all models trained with SMOTE is much better than the performance of models trained without SMOTE. The study shows the potential of machine learning methods for predicting all-cause mortality using cardiorespiratory fitness data.

## Background

Using data to make decisions and predications is not new. However, the nature of data availability is changing and the changes bring with them complexity in managing the volumes and analysis of these data. The marriage between mathematics and computer science is driven by the unique computational challenges of building predictive models from large data sets and getting into untapped hidden knowledge. Machine learning (ML) [1, 2] is a modern data analysis technique with the unique ability to learn and improve its performance without being explicitly programmed and without human instruction. The main goal of supervised ML classification algorithms [3] is to explain the dependent variable in terms of the independent variables. The algorithms get adjusted based on the training sample and the error signal. In general, conventional statistical techniques commonly rely on the process of hypothesis testing. This process is very user-driven where user specifies variables, functional form and type of interaction. Therefore, user intervention may influence resulting models. With ML techniques, the primary hypothesis is that there is a pattern (rather than an association) in the set of predictor variables that will identify the outcome. ML algorithms automatically scan and analyze all predictor variables in a way that prevents overlooking potentially important predictor variables even if it was unexpected. Therefore, it has been acknowledged as a powerful tool which dramatically changes the mode and accessibility of science, research and practice in all domains [4]. Medicine and Healthcare are no different [5–7].

The Henry Ford exercIse Testing (FIT) Project [8] is a retrospective cohort that included 69,985 patients who had undergone exercise cardiopulmonary treadmill stress testing at Henry Ford Health System in Detroit, MI from January 1, 1991- May 28, 2009. Briefly, the study population was limited to patients over 18 years of age at the time of stress testing and excluded patients undergoing modified or non- Bruce protocol [9] exercise stress tests. Information regarding a patient’s medical history, demographics, medications, cardiovascular disease risk factors were obtained at the time of testing by nurses and exercise physiologists, as well as searches through the electronic medical records. For the full details of The FIT Project, we refer to prior work by Al-Mallah et al. [8]. Several studies [10–13] have used conventional statistical techniques to predict various medical outcomes using the FIT project data. In general, ML is an exploratory process, where there is no one-model-fits-all solution. In particular, there is no model that is known to achieve the highest accuracy for all domains, problem types or datasets [14]. The best performing model varies from one problem to another based on the characteristics of the variables and observation. In this study, we evaluate and compare seven popular supervised ML algorithms in terms of its accuracy of prediction for mortality based on exercise capacity (e.g., fitness) data. In particular, we conducted experiments using the following ML techniques: Decision Tree (DT), Support Vector Machine (SVM), Artificial Neural Networks (ANN), Naïve Bayesian Classifier (BC), Bayesian Network (BN), K-Nearest Neighbor (KNN), and Random Forest (RF). We applied the 10-fold cross-validation evaluation method for all techniques where several evaluation metrics are compared and reported. The study shows the potential of machine learning methods for predicting all-cause mortality using cardiorespiratory fitness data.

## Methods

### Cohort study

In this study, we have excluded from the original registry of the FIT project the patients with known coronary artery disease (*n* = 10,190) or heart failure (*n* = 1162) at the time of the exercise test or with less than 10-year follow-up (*n* = 22; 890). Therefore, a total of 34,212 patients were included in this study. The baseline characteristics of the included cohort are shown in Table 1 and indicate a high prevalence of traditional risk factors for cardiovascular disease. After a follow-up duration of 10 years, a total of 3921 patients (11.5%) died as verified by the national social security death index. All included patients had a social security number and were accounted for. In this study, we have classified the patients into two categories: low risk of all-cause mortality (ACM) and high risk of ACM. In particular, patients were considered to have high risk for ACM if the predicted event rate is more than or equal to 3%.

**Table 1:** Baseline Characteristics for Included Study Cohort

Characteristic	Data (*n* = 34,212)
Age (years)^a^	54 ± 13
Male^b^	18,703 (55)
Race^b^	
White	23,801 (70)
Black	9768 (29)
Others	643 (1)
Body Mass Index (kg/m^2^)^a^	29.3 ± 5.8
Reason for Test^b^	
Chest Pain	17,547 (51)
Shortness of Breath	3307 (10)
Pre-Operation	781 (2)
Rule out Ischemia	3884 (11)
Stress Variables^a^	
Peak METS	9.2 ± 3.1
Resting Systolic Blood Pressure (mmHg)	132 ± 19
Resting Diastolic Blood Pressure (mmHg)	82 ± 11
Resting Heart rate (bpm)	74 ± 13
Peak Systolic Blood Pressure (mmHg)	183 ± 27
Peak Diastolic Blood Pressure (mmHg)	86 ± 14
Peak Heart Rate (bpm)	151 ± 21
Chronotropic incompetence^b^	6957 (23.3)
Past Medical History^b^	
Diabetes	5907(17)
Hypertension	20,534 (60)
Smoking	15,249 (43)
Family History of CAD	18,299 (51)
Medications Used^b^	
Diuretic Use	5743 (16)
Hypertensive medications	14,905 (42)
Diabetes medications	2432 (7)
Statin	4524 (13.2)
Aspirin	5752 (16.8)
Beta Blockers	5434 (15.9)
Calcium Channel Blockers	4638 (13.5)

*mmHg* millimeter mercury, *bpm* beat per minute, *CAD* coronary artery disease

All the data are presented as:

^a^Mean and standard deviation and

^b^frequencies and percentages

### Data Preprocessing

Data preprocessing is a crucial step in ML. Data that have not preprocessed carefully may lead to misleading prediction results. In our study, we have conducted the following preprocessing steps.

- *Outliers*: The dataset used has been preprocessed by removing outliers (values that deviate from the expected value for a specific attribute) using the statistical measure namely inter-quartile range (IQR) [15]. The authors in [1] compare different outlier detection methods on biomedical datasets. The results show that the IQR is the fastest method in detecting all outliers correctly. Since the dataset used in this study is nearly symmetric, its mean equals its median equals its midrange, then the IQR is a good choice for handling outliers. The IQR measure is used to preprocess and identify the outliers from the training dataset. The IQR finds the outliers from the dataset by identifying the data that is over ranging from the dataset. The IQR is evaluated as IQR = Q3-Q1 where Q3 and Q1 are the upper and lower quartiles, respectively. The number of records that are identified as outliers and has been removed is 808 records.
- *Missing values*: It has been noted that some attributes such as the Percentage of Achieved Heart Rate and Metabolic Equivalent (METS) have missing values. The missing data for such attributes has been handled by replacing the missing values by the attribute mean.

### Feature selection

The FIT project dataset includes 49 demographic and clinical variables.^1^ In general, it is a common case that a few or several of the variables used in ML predictive models are in fact not associated with the response. In practice, including such irrelevant variables leads to unnecessary complexity in the resulting model. Therefore, before developing our model, we utilized an automated R-based popular feature selection algorithm, information gain [16], to choose the most effective attributes in classifying the training data. In particular, this algorithm assesses the weight of each variable by evaluating the entropy gain with respect to the outcome, and then ranks the variables according to their weights. Only attributes with information gain >0 were subsequently used in model building.

### Sampling

One of the main issues we encountered with the dataset used in this study is that it is imbalanced. In particular, the dataset included 3946 records with class label Yes (high risk of all-cause mortality) and 30,985 records with class label No (low risk of all-cause mortality). In general, the predication accuracy is significantly affected with imbalanced data [17]. In practice, there are two ways to handle the imbalanced class problem. One way is assign distinct costs to examples in the training dataset [18]. The other way is to either oversampling the minority class or to under-sampling the majority class [19–22]. In order to handle the imbalanced dataset used in this study, we use Synthetic Minority Over-sampling (SMOTE) Technique [23]. It is an over-sampling technique in which the minority class is over-sampled by creating synthetic examples rather than by over-sampling with replacement. SMOTE selects the minority class samples and creates “synthetic” samples along the same line segment joining some or all *K* nearest neighbors belonging to the minority class [24, 25]. In other words, the oversampling is done as follows:

1. Take sample of the dataset and find its nearest neighbors
2. To create a synthetic data point, take the vector between a data point *P* in the sample dataset and one of *P’s* k-nearest neighbors.
3. Multiply this vector by a random number *x* which lies between 0 and 1.
4. Add this to *P* to create the new synthetic data point.

The percentage of SMOTE instances created in our experiment is 300% (11,838 records from the minority class).

### Machine learning classification techniques

In our experiments, we studied the following seven popular ML classification techniques: Decision Tree (DT), Support Vector Machine (SVM), Artificial Neural Networks (ANN), Naïve Bayesian Classifier (BC), Bayesian Network (BN), K-Nearest Neighbor (KNN) and Random Forest (RF). We explore the space of parameters and common variations for each machine learning algorithm as thoroughly as is computationally feasible.

*Decision Tree (DT)* [26] is a model that uses a tree-like graph to predict the value of a target variable by learning simple decision rules inferred from the data features. We use J48 decision tree algorithm (Weka implementation of C4.5 [27]). We tested the J48 classifier with confidence factor of 0.1, 0.25, 0.5, 0.75 and 1. The confidence factor parameter tests the effectiveness of post-pruning and lowering the confidence factor decreases the amount of post-pruning.

*Support Vector Machine (SVM)* [28] represents the instances as a set of points of 2 types in N dimensional place and generates a (N - 1) dimensional hyperplane to separate those points into 2 groups. SVM attempts to find a straight line that separates those points into 2 types and is situated as far as possible from all those points. Training the SVM is done using Sequential Minimal Optimization algorithm [2]. We used Weka implementation of SMO [29]. We tested SVM using polynomial, normalized polynomial, puk kernels and varied the complexity parameter {0.1, 10, and 30}. The value of the complexity parameter controls the tradeoff between fitting the training data and maximizing the separating margin.

*Artificial Neural Network (ANN)* [30] attempts to mimic the human brain in order to learn complex tasks. It is modeled as an interconnected group of nodes in a way that is similar to the vast network of neurons in the human brain. Each node of the network receives inputs from other nodes, combines them in some way, performs a generally nonlinear operation on the result and outputs the final result. We trained the Neural Networks with gradient descent backpropagation. We varied the number of hidden units {1, 2, 4, 8, 32} and the momentum {0,0.2,0.5,0.9}.

*Naïve Bayesian Classifier* [31] applies Bayes’ theorem [32] with the naive assumption of independence between every pair of features. We use Weka implementation of Multilayer Perceptron [33]. We try three different Weka options for handling continuous attributes: modeling them as a single normal, modeling them with kernel estimation, or discretizing them using supervised discretization. *Bayesian Network* [34] is designed for modeling under uncertainty where the nodes represent variables and arcs represent direct connections between them. BNs model allows probabilistic beliefs about the variables to be updated automatically as new information becomes available. We tried different search algorithms including K2 [33], Hill Climbing [35], Repeated Hill Climber, LAGD Hill Climbing, TAN [36], Tabu search [53] and Simulated annealing [37].

*K-Nearest Neighbors (KNN)* [38] identifies from the neighbors, K similar points in the training data that are closest to the test observation and classifies it by estimating the conditional probability of belonging to each class and choosing the class with the largest probability. We varied the number of k {1, 3, 5, 10} neighbors. We considered three distance functions: Euclidean distance, Manhattan distance and Minkowski distance.

*Random Forest (RF)* [39, 40] is a classification algorithm that works by forming multiple decision trees at training and at testing it outputs the class that is the mode of the classes (classification). Decision tree works by learning simple decision rules extracted from the data features. The deeper the tree, the more complex the decision rules and the fitter the model. Random decision forests overcome the problem of over fitting of the decision trees. We use Random Forest Weka implementation. We varied the forests to have 10, 50, and 100 trees. The size of the feature set considered at each split is 1, 2, 4, 8, and 12.

### Model evaluation and validation

In order to evaluate our models, we used the 10-fold cross-validation [39] evaluation method where the data are randomly partitioned into 10 mutually exclusive subsets {D_1_, D_2_, …, D_K_} with approximately equal size. The testing operation is then repeated 10 times where at the i^th^ evaluation iteration, the D_i_ subset is used as the test set and the others as the training set. In general, a main advantage of the 10-fold cross-validation evaluation method is that it has a lower variance than a single hold-out set evaluator. In particular, it reduces this variance by averaging over 10 different partitions, therefore, it is less sensitive to any partitioning bias on the training or testing data. For each iteration of the evaluation process, the following metrics are calculated:

- *Sensitivity*: True Positive recognition rate

Sensitivity = TP/TP + FN

- *Specificity*: True Negative recognition rate

Specificity = TN/TN + FP

- *Precision*: It represents the percentage of tuples that the classifier has labeled as positive are actually positive

Precision = TP/TP + FP

- *F-score*: It represents the harmonic mean of precision and sensitivity

*F-score* = 2 * TP / 2* TP + FP + FN

- Root Mean Squared Error (*RMSE*): It is defined as the square root of the mean square error that measures the difference between values predicted by the model and the actual values observed, where *y* ^′^ is a vector of *n* predictions and *y* is the vector of *n* observed (actual) values

$$ RMSD=\sqrt{\left(\frac{1}{n}\sum \limits_{i=1}^n{\left({y}_i^{\prime }-{y}_i\right)}^2\right)} $$

- *ROC*: Receiver Operating Characteristic (ROC) Curve [40] is a way to quantify the diagnostic value of a test over its whole range of possible cutoffs for classifying patients as positive vs. negative. In each possible cutoff, the true positive rate and false positive rate is calculated as the X and Y coordinates in the ROC Curve.

*True Positive (TP)* refers to the number of high risk patients who are classified as high risk, whereas *False Negative (FN)* refers to the number of high risk patients who are classified as low risk patients. On the other hand, *False Positive (FP)* refers to the number of low risk patients who are classified as high-risk patients and *False Negative (FN)* refers to the number of low risk patients who are classified as low risk patients. All results of the different metrics are then averaged to return the final result.

## Results

**Fig. 1:**
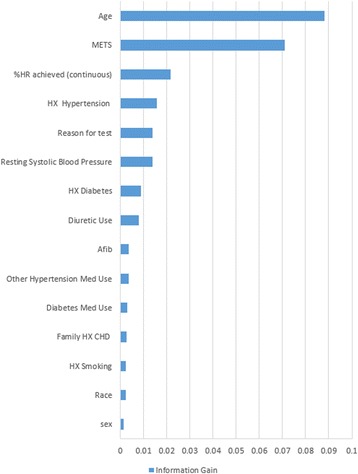
The ranking of the variables based on the outcome of the Feature Selection Process

As an outcome of the feature selection process, the ML models have been developed using only 15 variables where *Age*, *METS*, *Percentage HR achieved*, *HX Hypertension*, *Reason for test* are ranked as the top significant five variables. The full list of the outcome variables is presented in Fig. 1.

Tables 2 and 3 show the performance of the DT classifier, with confidence parameter (*Conf*) equals 0.1, 0.25, 0.5, 0.75 and 1, using sampling and without using sampling, respectively. The results show that the AUC increased as the confidence factor increased up to about 0.75 at a peak of 0.88 AUC using sampling and up to about 0.25 at a peak of 0.73 AUC without using sampling, after which the classifier exhibited effects of over-training. These effects are seen by a decrease in the AUC value with a confidence factor above 0.75 using sampling and above 0.25 without using sampling.

**Table 2:** Comparison of the performance of Decision Tree (DT) classifier with sampling using confidence parameter (*Conf*) equals 0.1, 0.25, 0.5, 0.75 and 1

	Conf = 0.1	Conf = 0.25	Conf = 0.5	Conf = 0.75	Conf = 1
Sensitivity	50.52%	55.71%	59.33%	59.95%	59.12%
Specificity	94.05%	64.97%	95.56%	96.05%	95.74%
Precision	55.69%	61.87%	67.08%	70.91%	68.52%
F-score	52.98%	58.63%	62.97%	64.97%	63.48%
RMSE	0.31	0.29	0.28	0.27	0.28
AUC	0.83	0.84	0.87	0.88	0.87

**Table 3:** Comparison of the performance of Decision Tree (DT) classifier without sampling using confidence parameter (*Conf*) equals 0.1, 0.25, 0.5, 0.75 and 1

	Conf = 0.1	Conf = 0.25	Conf = 0.5	Conf = 0.75	Conf = 1
Sensitivity	61.52%	54.43%	43.48%	36.11%	36.11%
Specificity	90.09%	90.51%	90.91%	90.95%	90.95%
Precision	18.21%	22.80%	28.16%	30.17%	30.17%
F-score	28.10%	32.14%	34.18%	32.87%	32.87%
RMSE	0.3	0.3	0.33	0.35	0.35
AUC	0.72	0.73	0.69	0.65	0.65

The results of the SVM classifier using sampling and without using sampling are reported in Tables 4 and 5, respectively. Different kernels (polynomial kernel, normalized polynomial kernel and puk kernel) and complexity parameters (*C*) (0.1, 10 and 30) are tested. The results show that the AUC increased as the complexity parameter increased up to 30 using sampling. In addition, the SVM using puk kernel outperforms the SVM using other kernels achieving AUC of 0.80 using sampling and 0.59 without using sampling with complexity parameter *C* = 30.

**Table 4:** Comparison of the performance of Support Vector Machine (SVM) classifier with sampling using polynomial, normalized polynomial and puk kernels using complexity parameters 0.1, 10 and 30

	Polynomial			Normalized Polynomial			Puk		
	C = 0.1	C = 10	C = 30	C = 0.1	C = 10	C = 30	C = 0.1	C = 10	C = 30
Sensitivity	36.18%	36.18%	36.18%	100%	95.10%	65.10%	47.38%	81.94%	80.26%
Specificity	94.37%	94.37%	94.37%	88.31%	88.79%	88.85%	88.58%	94.13%	95.19%
Precision	61.46%	61.41%	61.41%	0.02%	33.67%	5.62%	6.33%	53.64%	62.63%
F-score	45.55%	45.53%	45.53%	0.05%	49.73%	10.35%	11.17%	64.84%	70.36%
RMSE	0.41	0.42	0.42	0.34	0.34	0.34	0.35	0.26	0.25
AUC	0.74	0.74	0.74	0.5	0.52	0.53	0.53	0.76	0.8

**Table 5:** Comparison of the performance of Support Vector Machine (SVM) classifier without sampling using polynomial, normalized polynomial and puk kernels using complexity parameters 0.1, 10 and 30

	Polynomial			Normalized Polynomial			Puk		
	C = 0.1	C = 10	C = 30	C = 0.1	C = 10	C = 30	C = 0.1	C = 10	C = 30
Sensitivity	0%	0%	0%	0%	0%	56.59%	0%	37.90%	39.78%
Specificity	88.30%	88.30%	88.30%	88.30%	88.30%	88.83%	88.30%	90.22%	87.65%
Precision	0%	0%	0%	0%	0.00%	5.55%	0%	22.11%	18.03%
F-score	0%	0%	0%	0%	0.00%	10.11%	0%	27.92%	24.81%
RMSE	0.34	0.34	0.34	0.34	0.34	0.34	0.34	0.37	0.47
AUC	0.50	0.50	0.50	0.5	0.5	0.52	0.5	0.58	0.59

Tables 6 and 7 show the performance of Neural Networks with gradient descent backpropagation using hidden units *H* = {1, 2, 4, 8, 32} and the momentum *M* = {0, 0.2, 0.5, 0.9} using sampling and without using sampling, respectively. The number of hidden units and momentum rate that gives better AUC value is considered here. For neural networks, the highest performance is achieved when *H* = 4 and *M* = 0.5 for the case of using sampling (AUC = 0.82) while when *H* = 8 and *M* = 0 for the case of not using sampling (AUC = 0.80).

**Table 6:** Comparison of the performance of Artificial Neural Networks (ANN) classifier with gradient descent backpropagation using hidden units {1, 2, 4, 8, 32} and the momentum {0,0.2,0.5,0.9} using sampling

	H = 1				H = 2				H = 4				H = 8				H = 32			
	M = 0	M = 0.2	M = 0.5	M = 0.9	M = 0	M = 0.2	M = 0.5	M = 0.9	M = 0	M = 0.2	M = 0.5	M = 0.9	M = 0	M = 0.2	M = 0.5	M = 0.9	M = 0	M = 0.2	M = 0.5	M = 0.9
Sensitivity	–	54.27%	–	45.83%	57.63%	56.81%	56.15%	50.86%	55.79%	55.61%	55.89%	47.38%	54.87%	51.87%	50.94%	51.02%	43.82%	45.96%	44.17%	58.70%
Specificity	88.01%	88.35%	88.01%	89.10%	90.51%	90.61%	90.78%	90.30%	89.82%	90.13%	90.43%	89.67%	90.31%	90.29%	90.39%	89.77%	90.43%	90.60%	90.19%	88.09%
Precision	0	3.60%	0	11.90%	24.93%	26.00%	27.70%	23.60%	18.47%	21.47%	24.37%	17.77%	23.30%	23.57%	24.50%	18.33%	25.87%	27.13%	23.37%	0.90%
F-score	0	6.75%	0	18.89%	34.81%	35.67%	37.10%	32.24%	27.75%	30.98%	33.94%	25.84%	32.71%	32.41%	33.09%	26.97%	32.53%	34.12%	30.56%	1.77%
RMSE	0.30	0.30	0.30	0.30	0.29	0.29	0.29	0.30	0.29	0.29	0.29	0.30	0.30	0.30	0.30	0.32	0.32	0.32	0.32	0.35
AUC	0.77	0.76	0.74	0.72	0.8	0.79	0.77	0.72	0.8	0.81	0.82	0.78	0.81	0.81	0.81	0.68	0.77	0.77	0.78	0.52

**Table 7:** Comparison of the performance of Artificial Neural Networks (ANN) classifier with gradient descent backpropagation using hidden units {1, 2, 4, 8, 32} and the momentum {0,0.2,0.5,0.9} without using sampling

	H = 1				H = 2				H = 4				H = 8				H = 32			
	M = 0	M = 0.2	M = 0.5	M = 0.9	M = 0	M = 0.2	M = 0.5	M = 0.9	M = 0	M = 0.2	M = 0.5	M = 0.9	M = 0	M = 0.2	M = 0.5	M = 0.9	M = 0	M = 0.2	M = 0.5	M = 0.9
Sensitivity	–	–	42.30%	–	52.65%	52.72%	52.40%	61.99%	49.67%	52.10%	50.99%	47.50%	51.16%	49.19%	51.93%	51.89%	42.69%	40.05%	42.31%	66.67%
Specificity	88.30%	88.30%	90.62%	88.30%	91.37%	91.32%	91.42%	89.29%	90.90%	89.96%	90.59%	91.64%	90.89%	90.79%	90.56%	89.37%	90.83%	90.98%	91.07%	88.38%
Precision	0	0	25.43%	0	31.39%	30.86%	31.84%	10.14%	27.18%	17.51%	24%	34.57%	26.94%	26.12%	23.54%	11.51%	27.46%	29.55%	29.93%	0.77%
F-score	0	0	31.76%	0	39.33%	38.93%	39.61%	17.43%	35.13%	26.21%	32.63%	40.02%	35.29%	34.13%	32.40%	18.84%	33.43%	34.00%	35.06%	1.51%
RMSE	0.29	0.30	0.30	0.30	0.29	0.29	0.29	0.30	0.29	0.29	0.30	0.30	0.30	0.30	0.30	0.31	0.33	0.33	0.33	0.34
AUC	0.79	0.77	0.76	0.73	0.78	0.78	0.79	0.79	0.78	0.78	0.79	0.79	0.80	0.80	0.80	0.79	0.77	0.76	0.76	0.50

The performance of the Naïve Bayesian Classifier using sampling and without using sampling is reported in Tables 8 and 9, respectively. Three different Weka options for handling continuous attributes are explored (single normal, kernel estimation and supervised discretization). Results show that BC using supervised discretization achieves the highest AUC value of 0.82 using sampling and without using sampling. The performance results of the Bayesian Network classifier with different search algorithms (K2, Hill Climbing, Repeated Hill Climber, LAGD Hill Climbing, TAN, Tabu and Simulated Annealing) using sampling and without using sampling are reported in Tables 10 and 11, respectively. Bayesian Network classifier using Tan search algorithm achieves the highest AUC value of 0.84 using Sampling and 0.83 without using sampling.

**Table 8:** Comparison of the performance of Naïve Bayesian classifier (BC) using three different Weka options for handling continuous attributes: single normal, kernel estimation and supervised discretization using Sampling

	Single Normal	kernel Estimation	Supervised Discretization
Sensitivity	35.32%	40.90%	37.41%
Specificity	93.26%	92.37%	93.32%
Precision	52.34%	42.70%	52.20%
F-score	42.18%	41.78%	43.59%
RMSE	0.35	0.32	0.34
AUC	0.81	0.81	0.82

**Table 9:** Comparison of the performance of Naïve Bayesian classifier (BC) using three different Weka options for handling continuous attributes: single normal, kernel estimation and supervised discretization without using Sampling

	Single Normal	kernel Estimation	Supervised Discretization
Sensitivity	35.73%	41.25%	37.71%
Specificity	93.22%	92.17%	93.23%
Precision	51.89%	40.79%	51.32%
F-score	42.32%	41.02%	43.47%
RMSE	0.35	0.32	0.34
AUC	0.81	0.81	0.82

**Table 10:** Comparison of the performance of Bayesian Network classifier (BN) using different search algorithms: K2, Hill Climbing, Repeated Hill Climber, LAGD Hill Climbing, TAN, Tabu and Simulated Annealing using Sampling

	K2	Hill Climbing	Repeated Hill Climber	LAGD Hill Climbing	TAN	Tabu	Simulated Annealing
Sensitivity	37.44%	37.44%	37.44%	47.65%	60.07%	37.59%	55.20%
Specificity	93.31%	93.31%	93.31%	91.55%	91.02%	93.20%	91.23%
Precision	52.11%	52.11%	52.11%	33.76%	27.32%	51.10%	29.71%
F-score	43.57%	43.57%	43.57%	39.52%	37.56%	43.31%	38.63%
RMSE	0.34	0.34	0.34	0.34	0.28	0.34	0.29
AUC	0.82	0.82	0.82	0.81	0.84	0.81	0.84

**Table 11:** Comparison of the performance of Bayesian Network classifier (BN) using different search algorithms: K2, Hill Climbing, Repeated Hill Climber, LAGD Hill Climbing, TAN, Tabu and Simulated Annealing without using Sampling

	K2	Hill Climbing	Repeated Hill Climber	LAGD Hill Climbing	TAN	Tabu	Simulated Annealing
Sensitivity	37.70%	37.70%	37.70%	48.11%	57.09%	37.94%	53.65%
Specificity	93.21%	93.21%	93.21%	91.44%	90.71%	93.19%	90.97%
Precision	51.20%	51.20%	51.20%	32.63%	24.57%	50.89%	27.44%
F-score	43.42%	43.42%	43.42%	38.89%	34.35%	43.47%	36.31%
RMSE	0.34	0.34	0.34	0.34	0.34	0.3	0.3
AUC	0.82	0.82	0.82	0.81	0.83	0.81	0.82

Tables 12 and 13 report the performance of the KNN classifier, with different values of k {1, 3, 5, 10} neighbors, and using sampling and without using sampling. In our experiments, we used different distance functions; Euclidean distance, Manhattan distance and Minkowski distance. The results show that the KNN classifier using sampling has its best performance (AUC = 0.88) with K value equals 1 using any of the three distance functions while the KNN classifier without using sampling has its best performance (AUC = 0.74) with K value equals 10 using any of the three distance functions.

**Table 12:** Comparison of the performance K-Nearest Neighbor classifier (KNN) using different values of k {1, 3, 5, 10} neighbors and using different distance functions; Euclidean distance, Manhattan distance and Minkowski distance using sampling

	Euclidean distance				Manhattan Distance				Minkowski Distance			
	K = 1	K = 3	K = 5	K = 10	K = 1	K = 3	K = 5	K = 10	K = 1	K = 3	K = 5	K = 10
Sensitivity	78.43%	65.61%	64.17%	50.00%	78.29%	65.66%	65.68%	61.23%	78.43%	65.61%	64.17%	59.23%
Specificity	96.98%	91.74%	90.53%	89.84%	97.05%	91.80%	90.60%	89.91%	96.98%	91.74%	90.53%	89.84%
Precision	77.18%	33.64%	22.32%	11.50%	77.73%	34.16%	22.94%	16.44%	77.18%	33.64%	22.32%	15.89%
F-score	77.80%	44.47%	33.12%	18.70%	78.01%	44.94%	34.01%	25.91%	77.80%	44.47%	33.12%	25.05%
RMSE	0.23	0.27	0.28	0.29	0.23	0.27	0.28	0.29	0.23	0.27	0.28	0.29
AUC	0.88	0.86	0.85	0.84	0.87	0.86	0.85	0.84	0.87	0.86	0.85	0.84

The results show that the value 1 for the K parameter achieves the highest AUC (0.88) using Euclidean distance

**Table 13:** Comparison of the performance K-Nearest Neighbor classifier (KNN) using different values of k {1, 3, 5, 10} neighbors and using different distance functions; Euclidean distance, Manhattan distance and Minkowski distance without using sampling

	Euclidean distance				Manhattan Distance				Minkowski Distance			
	K = 1	K = 3	K = 5	K = 10	K = 1	K = 3	K = 5	K = 10	K = 1	K = 3	K = 5	K = 10
Sensitivity	28.06%	38.19%	42.44%	46.78%	28.54%	38.21%	42.96%	47.59%	28.06%	38.19%	42.44%	28.06%
Specificity	90.24%	89.88%	89.50%	89.31%	90.28%	89.87%	89.49%	89.31%	90.24%	89.88%	89.50%	90.24%
Precision	25.36%	18.37%	13.64%	11.12%	25.62%	18.18%	13.42%	11.12%	25.36%	18.37%	13.64%	25.36%
F-score	26.64%	24.81%	20.64%	17.97%	27.00%	24.64%	20.45%	18.03%	26.64%	24.81%	20.64%	26.64%
RMSE	0.4	0.33	0.32	0.3	0.4	0.33	0.32	0.31	0.4	0.33	0.32	0.4
AUC	0.58	0.66	0.7	0.74	0.59	0.67	0.7	0.74	0.58	0.66	0.7	0.58

The results show that the value 10 for the K parameter achieves the highest AUC (0.74) using Euclidean distance

Tables 14 and 15 report the performance of the Random Forest (RF) classifier using 10, 50 and 100 trees. The size of the feature set (*F*) considered at each split is 1, 2, 4, 8, and 12. The results show that the highest AUC (0.97) is achieved using a forest of 50 trees with a feature set of 1, 2, 4, 8 or 12 using sampling whereas the highest AUC (0.82) is achieved using a forest of 100 trees with a feature set of 4.

**Table 14:** Comparison of the performance of Random Forest (RF) classifier having 10, 50 and 100 trees with different feature set considered at each split (1, 2, 4, 8, and 12) using sampling

	No. of tree =10					No. of tree =50					No. of tree =100				
	F = 1	F = 2	F = 4	F = 8	F = 12	F = 1	F = 2	F = 4	F = 8	F = 12	F = 1	F = 2	F = 4	F = 8	F = 12
Sensitivity	90.62%	91.01%	89.46%	87.40%	86.90%	96.07%	95.47%	94.67%	93.63%	93.14%	96.73%	95.97%	94.85%	93.95%	93.59%
Specificity	96.49%	96.56%	96.67%	96.79%	96.83%	96.84%	96.85%	97.06%	97.11%	97.15%	96.88%	96.88%	97.04%	97.19%	97.18%
Precision	72.78%	73.40%	74.28%	75.31%	75.67%	75.50%	75.57%	77.27%	77.73%	77.99%	75.74%	75.81%	77.08%	78.35%	78.28%
F-score	80.72%	81.26%	81.17%	80.90%	80.90%	84.55%	84.36%	85.09%	84.94%	84.90%	84.96%	84.71%	85.05%	85.44%	85.25%
AUC	80.72	81.26	81.17	80.90	80.90	0.97	0.97	0.97	0.97	0.97	0.97	0.97	0.97	0.97	0.97
RMSE	0.2	0.19	0.2	0.2	0.2	0.18	0.18	0.18	0.18	0.18	0.18	0.18	0.18	0.18	0.18

**Table 15:** Comparison of the performance of Random Forest (RF) classifier having 10, 50 and 100 trees with different feature set considered at each split (1, 2, 4, 8, and 12) without using sampling

	No. of tree =10					No. of tree =50					No. of tree =100				
	F = 1	F = 2	F = 4	F = 8	F = 12	F = 1	F = 2	F = 4	F = 8	F = 12	F = 1	F = 2	F = 4	F = 8	F = 12
Sensitivity	45.82%	47.20%	48.35%	46.64%	45.44%	56.62%	56.34%	57.33%	55.84%	54.51%	58.39%	59.87%	59.09%	56.56%	54.41%
Specificity	90.03%	90.23%	90.58%	90.83%	90.85%	89.60%	89.81%	90.29%	90.48%	90.57%	89.50%	89.81%	90.21%	90.45%	90.48%
Precision	18.90%	20.77%	24.19%	26.91%	27.30%	13.61%	15.74%	20.41%	22.42%	23.42%	12.49%	15.53%	19.52%	22.08%	22.56%
F-score	26.76%	28.84%	32.24%	34.13%	34.11%	21.95%	24.61%	30.10%	31.99%	32.76%	20.58%	24.66%	29.35%	31.76%	31.90%
RMSE	0.3	0.3	0.3	0.3	0.31	0.29	0.29	0.29	0.29	0.30	0.29	0.29	0.29	0.29	0.29
AUC	0.76	0.77	0.77	0.77	0.76	0.81	0.81	0.81	0.81	0.80	0.81	0.81	0.82	0.81	0.81

**Fig. 2:**
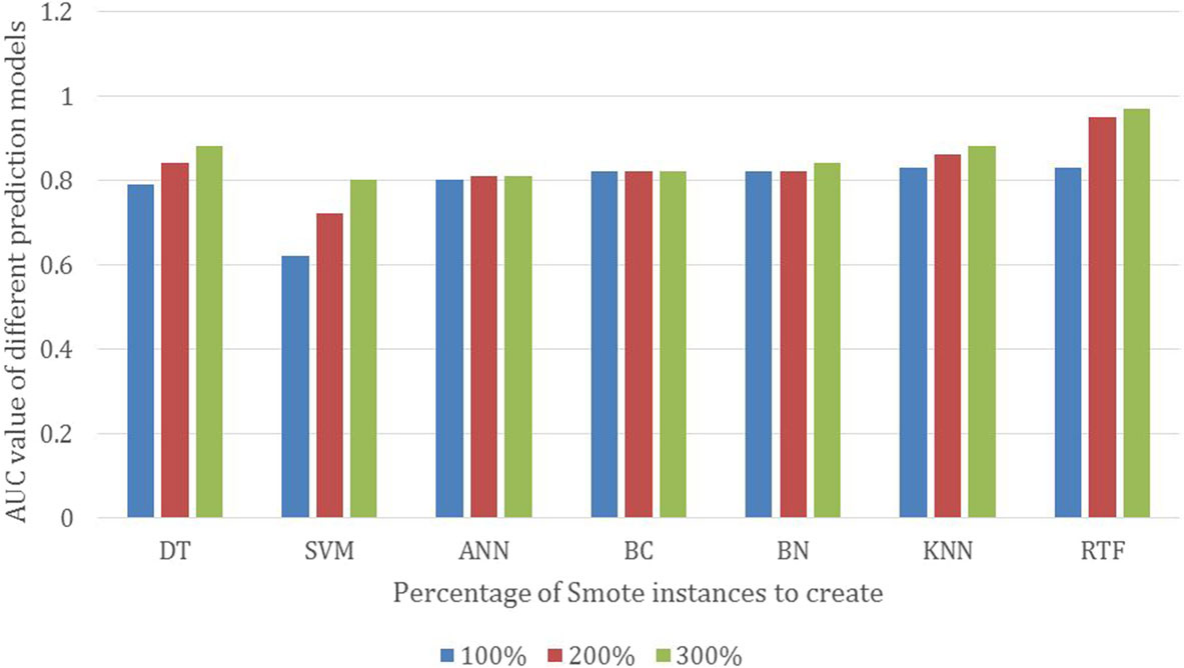
AUC of different models with different percentage of synthetic examples created using SMOTE

We compared the impact of using different percentage of synthetic examples of the class “yes” (patients who are considered to have high risk for ACM). Figure 2 shows the area under the curve of seven different machine learning models trained using Decision Tree (DT), Support Vector Machine (SVM), Artificial Neural Networks (ANN), Naïve Bayesian Classifier (BC), Bayesian Network (BN), K-Nearest Neighbor (KNN) and Radom Forest (RF). All the models have been evaluated using datasets with 100%, 200% and 300% of synthetic example created using the SMOTE sampling technique on the training dataset and evaluated using 10-fold cross validation. The results show that increasing the percentage of synthetic examples improves the prediction accuracy for all models except for the BC. For example, the SVM model achieves AUC of 0.62 using the sampled dataset with 100% synthetic examples compared to 0.72 using the sampled dataset with 200% synthetic examples. Increasing the percentage of synthetic examples to 300% improves the AUC of the BN to achieve 0.8. The performance of KNN, DT and RTF models using SMOTE has shown great improvement. The RF has shown the best improvement using SMOTE achieving 0.83 using 100% synthetic examples compared to 0.95 and 0.97 using 200% and 300% synthetic examples respectively. In our experiments, further increasing the synthetic examples to 400% and 500% did not show any improvement in the performance of the prediction models.

**Fig. 3:**
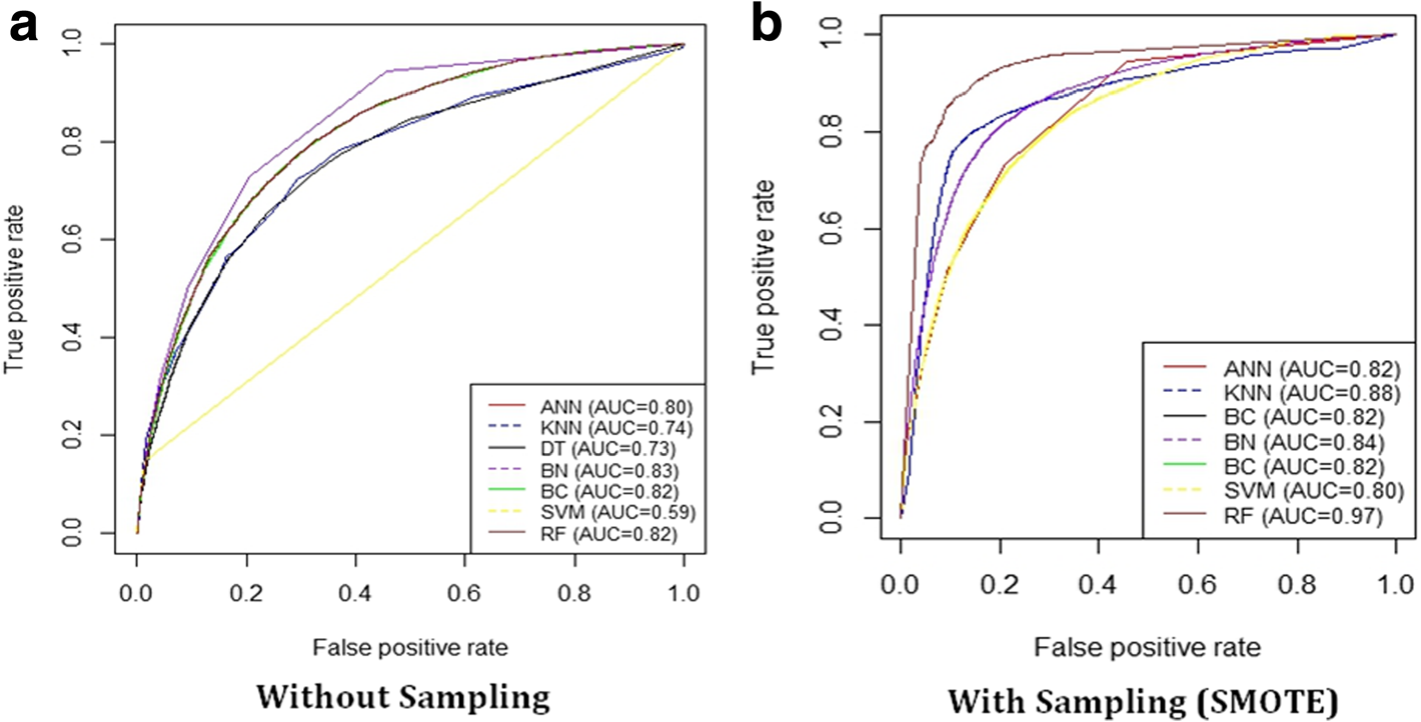
The ROC curves of the different machine learning classification models. The models are: Decision Tree (DT), Support Vector Machine (SVM), Artificial Neural Networks (ANN), Naïve Bayesian Classifier (BC), Bayesian Network (BN) and K-Nearest Neighbor (KNN). The results show that without using the SMOTE sampling method (**a**), BC and BN achieves the highest AUC (0.81) while with using the SMOTE sampling method (**b**), the KNN model achieves the highest AUC (0.94)

In order to evaluate the impact of using the SMOTE sampling techniques in handling the problem of the imbalanced dataset, we build different prediction models with and without SMOTE. Tables 16 and 17 show the prediction performance of different prediction models using various evaluation metrics without and with the SMOTE sampling technique (300%), respectively. For each metric (row), we highlighted the highest value in bold font and underlined the lowest value. As shown in Tables 16 and 17, after applying the 10-fold cross-validation on the training dataset, the AUC and sensitivity for all models used SMOTE have been significantly improved over the training results without SMOTE except for the BC. In addition, the performance of each model can differ from one metric to another. In general, the Random Forest (RF) classifier using SMOTE sampling achieves the best performance improvement. In particular, it achieves the best performance in terms of Sensitivity (95.07%), RMSE (0.18), F-Score (84.55%) and AUC (0.97). However, the same model without using SMOTE achieves Sensitivity of (59.09%), RMSE of (0.29), F-Score (29.35%) and AUC of (0.82). The KNN models using SMOTE achieves the best performance in terms of Specificity (96.98%) and Precision (77.18%). The KNN model without SMOTE achieves Specificity of 89.31%% and Precision of 11.12%. This improved performance of the prediction models is due to the imbalanced data size. It is noted that all the models with SMOTE achieve a more balanced sensitivity. Figure 3(a) and (b) illustrates the ROC curves for the different ML models with and without using SMOTE, respectively.

**Table 16:** Comparison of the performance of the different classification models without using the SMOTE sampling method

	DT	SVM	ANN	BC	BN	KNN	RF
Sensitivity	54.43%	39.78%	52.65%	37.71%	57.09%	46.78%	59.09%
Specificity	90.51%	87.65%	91.37%	**93.23%**	90.71%	89.31%	90.21%
Precision	22.80%	18.03%	31.39%	**51.32%**	24.57%	11.12%	19.52%
F-score	32.14%	24.81%	39.33%	**43.47%**	34.35%	17.97%	29.35%
RMSE	0.3	**0.47**	0.29	0.34	0.34	0.3	0.29
AUC	0.73	0.59	0.80	0.82	**0.83**	0.74	0.82

The models are: Decision Tree (DT), Support Vector Machine (SVM), Artificial Neural Networks (ANN), Naïve Bayesian Classifier (BC), Bayesian Network (BN), K-Nearest Neighbor (KNN) and Random Forest (RF). The results of this experiment show that BN achieves the highest AUC (0.83). The BC model achieves the highest precision (51.32%) and the highest specificity (93.32%)

**Table 17:** Comparison of the performance of the different classification models using the SMOTE sampling methods. The models are: Decision Tree (DT), Support Vector Machine (SVM), Artificial Neural Networks (ANN), Naïve Bayesian Classifier (BC), Bayesian Network (BN), K-Nearest Neighbor (KNN) and Random Forest (RF)

	DT	SVM	ANN	BC	BN	KNN	RF
Sensitivity	59.95%	80.26%	55.89%	37.41%	60.07%	78.43%	**96.07%**
Specificity	96.05%	95.19%	90.43%	93.32%	91.02%	**96.98%**	96.84%
Precision	70.91%	62.63%	24.37%	52.20%	27.32%	**77.18%**	75.50%
F-score	64.97%	70.36%	33.94%	43.59%	37.56%	77.80%	**84.55%**
RMSE	0.27	0.25	0.29	**0.34**	0.28	0.23	0.18
AUC	0.88	0.8	0.82	0.82	0.84	0.88	**0.97**

The results of this experiment show that the RF model achieves the highest AUC (0.97), the lowest RMSE (0.18) and the highest sensitivity (94.65%)

## Discussion

Using machine learning methods to predict different medical outcomes (e.g., diabetics, hypertension and death) from medical datasets is gaining an increasing attention in the medical domain. This study is designed to take advantage of the unique opportunity provided by our access to a large and rich clinical research dataset, a total of 34,212 patients, collected by the FIT project to investigate the relative performance of various machine learning classification methods for predicting all-cause mortality (ACM) using medical records of cardiorespiratory fitness. The large number of attributes of the dataset, 49 attributes, is used to uncover new potential predictors of ACM. To the best of our knowledge, this is the first study that compares the performance of ML model for predicting ACM using cardiorespiratory fitness data. We have evaluated seven models trained with and without SMOTE using various evaluation metrics.

Knuiman et al. [41] presented an empirical comparison of four different techniques for estimating the risk of death using mortality follow-up data on 1701 men. The four techniques used are binary tree, logistic regression, survival tree and Cox regression. The Cox regression outperformed the other three techniques achieving area under the AUC of 0.78 followed by logistic regression (AUC = 0.72), survival tree (AUC = 0.71) and binary tree (AUC = 0.66), respectively. Vomlel et al. [42] presented a predictive model for mortality using five different machine learning techniques on a data of 603 patients from University Hospital in Olomouc. The machine learning techniques used are logistic regression, decision tree, Naive Bayes classifier, Artificial Neural Network and Bayesian Network classifier. Using 10- fold cross validation logistic regression achieves the highest area under curve of 0.82, whereas the decision tree has the lowest AUC value of 0.61. Allyn et al. [43] compared the performance of logistic regression model and different machine learning models to predict the mortality in-hospital after elective cardiac surgery. The study includes database of 6520 patients from December 2005 to December 2012, from a cardiac surgical center at University Hospital. Five different machine learning models have been evaluated: logistic regression, gradient boosting machine, random forest, support vector machine and naive bayes. The area under the ROC curve for the machine learning model (AUC = 0.795) was significantly higher than the logistic regression model (AUC = 0.742). Taylor et al. [44] studied the prediction of mortality of 4676 patients with sepsis at the emergency department using logistic regression and machine learning model. The machine learning model (AUC 0.86) outperforms the logistic regression model (AUC 0.76). Sherri [45] studied the Physical Performance and Age-Related Changes in Sonomans (SPPARCS) to predict death among 2066 residents of Sonoma, California over the period between 1993 and 1995. In this study, a super learner has been used for death prediction. A super learner is an ensembling machine learning approach that combines multiple machine learning algorithms into a single algorithm and returns a prediction function with the best cross-validated mean squared error. The super learner outperforms all single algorithms in the collection of algorithms, although its performance was quite similar to that of some algorithms. Super learner outperformed the worst algorithm (neural networks) by 44% with respect to estimated cross-validated mean squared error. In principle, the datasets of both studies (Knuiman et al. [41] and Allyn et al. [43]) are considered to be relatively small in comparison to the number of patients for our dataset. In general, in Machine Learning, the bigger the size of the dataset, the higher the accuracy and robustness of the developed prediction models. In these studies, the highest AUC achieved by the developed prediction models is 0.86. In our experiments, the Random Forest (RF) model using SMOTE sampling achieved AUC of 0.97which significantly outperform the models of both studies.

Sullivan et al. [46] investigated the literature related to the comparisons made between established risk prediction models for perioperative mortality used in the setting of cardiac surgery. Meta-analysis was conducted to calculate a summary estimate of the difference in AUCs between models. The comparisons include 22 studies. The authors noted that all he investigated studies relied on relatively small datasets. This highlights the strengths and uniqueness of our study which is relying on large datasets reflected on the number of patients and the number of variables.

In general, an important observation from the results of our experiments is that for all metrics, the results show that it is not necessarily that the complex ML models (e.g., Support Vector Machine (SVM), Artificial Neural Networks (ANN)) can always outperform simpler models (e.g., Decision Tree (DT) model [47]). In particular, the Decision Tree (DT) model has been outperforming the complex models in terms of all evaluation metrics. The RF and KNN classifiers are considered to be less complex than SVM and ANN. However, it achieved the best performance for all metrics for model trained using SMOTE. In general, KNN is a non-linear classifier, therefore, it tends to perform very well with a lot of data points. It is also very sensitive to bad features (variables). Therefore, effective feature selection [27] is an important step before using the KNN classifier and tends to improve its results. The Decision Tree (DT) model benefits from the feature selection and removing colinear variables steps as well. In general, decision trees do not require any assumptions of linearity in the data and thus they work well for nonlinearly related variables.

On the other hand, the SVM model tends to perform well in high-dimensioned classification problems that may have over hundreds of thousands of dimensions, which is not the case of this study. In addition, the SVM model does not tend to perform well if the classes of the problem are strongly overlapping. In general, parametric models (e.g., SVM, Bayesian Network) can suffer from remembering local groupings as by their nature they summarize information in some way. ANN can usually outperform other methods if the dataset is very large and if the structure of the data is complex (e.g., they have many layers). This is an advantage for the KNN classifier which makes the least number of assumptions regarding the input data.

The results also show that the performance of the KNN and ANN classifiers, similar to the other models, can be very sensitive for the values of its parameters and thus these parameters need to be carefully explored and tuned in order to reach an adequate configuration. For example, the results show that setting the K parameter to the value of 1 achieves the best performance for all the evaluation metrics. For example, for K = 1, the model achieves AUC of (0.94) while for K = 3, 5 and 10, the model achieves the accuracy of (0.93), (0.91) and (0.90), respectively. In general, increasing the value of the K parameter has a mostly negative impact on the performance of the classifier for all metrics. The risk of model overfilling by using a low K value has been overcome by using the 10-fold cross-validation evaluation method. However, clearly, the optimal value of the *K* parameter can significantly differ from one problem to another.

## Conclusion

ML techniques have shown solid prediction capabilities in various application domains including medicine and healthcare. In this study, we presented an evaluation and comparison of seven popular ML techniques on predicating all-cause mortality (ACM) using medical records of Cardiorespiratory Fitness for the Henry Ford Testing (FIT) Project. The results show that various ML techniques can significantly vary in terms of its performance for the different evaluation metrics. It is also not necessarily that the more complex the ML model, the more prediction accuracy can be achieved. Simpler models can perform better in some cases as well. Therefore, there is no one-size-fits-all model that can be well performing for all domains or datasets. Each problem and dataset need to be carefully evaluated, modeled and studied in order to reach an effective predictive model design. The results have also shown that it is critical to carefully explore and evaluate the performance of the ML models using various tuned values for their parameters. These results confirm the explorative nature of the ML process that requires iterative and explorative experiments in order to discover the model design that can achieve the target accuracy.

## Abbreviations

ACM: All-cause mortality
ANN: Artificial Neural Networks
BC: Naïve Bayesian Classifier
BN: Bayesian Network
CRF: Cardiorespiratory Fitness.
DT: Decision Tree
KNN: K-Nearest Neighbor
ML: Machine Learning
RF: Random Forest.
SMOTE: Synthetic Minority Over-Sampling Technique (SMOTE)
SVM: Support Vector Machine

## Footnotes

1 The detailed descriptions of the variables of the dataset are available on the appendix of the article.
